# Lactate-to-Albumin Ratio (LAR) as a Predictor of All-Cause Mortality in Patients With Myocardial Infarction: A Systematic Review and Meta-Analysis

**DOI:** 10.7759/cureus.82166

**Published:** 2025-04-13

**Authors:** Mujahed Ul Islam, Uday Gollapinni, Shabab Ul Hassan, Muhammad Mohsin Saleem, Harshith R Venkannagari, Sandipkumar S Chaudhari, Moaz Mohsin, Areeba Khan

**Affiliations:** 1 Infectious Diseases, University Hospitals of North Midlands, Stoke-on-Trent, GBR; 2 Acute Medicine, Royal Stoke University Hospital, Stoke-on-Trent, GBR; 3 Internal Medicine, University Hospitals of North Midlands, Stoke-on-Trent, GBR; 4 Medicine, University Hospitals of North Midlands, Stoke-on-Trent, GBR; 5 Emergency, University Hospitals of North Midlands, Stoke-on-Trent, GBR; 6 Cardiothoracic Surgery, The University of Alabama at Birmingham, Birmingham, USA; 7 Family Medicine, University of North Dakota School of Medicine and Health Sciences, Fargo, USA; 8 Critical Care Medicine, United Medical and Dental College, Karachi, PAK

**Keywords:** lactate to albumin ratio, mortality, myocardial infarction, prognostic biomarker, risk stratification

## Abstract

Myocardial infarction (MI) remains a significant global health challenge, necessitating innovative approaches for early risk stratification. This systematic review and meta-analysis investigated the prognostic value of the lactate-to-albumin ratio (LAR) in predicting all-cause mortality among MI patients. A comprehensive literature search was conducted across PubMed, Embase, Web of Science, and Google Scholar, encompassing studies published until March 10, 2025. The meta-analysis included five studies involving 8,408 subjects with MI, all conducted in China between 2022 and 2024. Studies were selected based on predefined eligibility criteria, focusing on observational cohort and case-control designs, with LAR measured at admission and mortality outcomes reported. Pooled analysis revealed that patients with high LAR experienced approximately twice the risk of all-cause mortality compared to those with low LAR (hazard ratio: 2.08, 95% confidence interval: 1.70-2.54,p < 0.001). Significant heterogeneity was observed among studies (I^2^: 75%), which may be attributed to variations in patient populations and measurement methodologies. Despite limitations such as retrospective study designs and potential selection bias, the findings suggest LAR as a promising, accessible biomarker for early risk stratification in MI patients. Future research should focus on prospective studies to validate these results, establish standardized measurement protocols, and explore the underlying physiological mechanisms linking LAR to adverse outcomes.

## Introduction and background

Myocardial infarction (MI) remains a leading cause of morbidity and mortality worldwide, despite substantial advances in early diagnosis, reperfusion strategies, and pharmacological interventions [1]. Accurate risk stratification at the time of presentation is essential for guiding treatment decisions and improving clinical outcomes. While traditional biomarkers - such as cardiac troponins, creatine kinase-MB, and natriuretic peptides - are well established for diagnosis and prognostication, emerging biomarkers that reflect broader pathophysiological processes may offer additional value [2].

Among these, the lactate-to-albumin ratio (LAR) has recently garnered attention as a composite marker that integrates metabolic and inflammatory status [3]. Lactate is a well-known marker of tissue hypoxia and impaired perfusion, commonly elevated in the context of cardiogenic shock and ischemia [4]. In contrast, albumin is a negative acute-phase protein that reflects nutritional status and systemic inflammation, and hypoalbuminemia has been associated with poor outcomes in several cardiovascular conditions, including MI [5]. Although both biomarkers individually convey prognostic information, each has limitations: lactate levels can be transiently elevated due to non-cardiac causes, while albumin may be influenced by chronic comorbidities or hepatic function [6,7]. By combining these two parameters, LAR may offer a more stable and comprehensive reflection of a patient’s overall physiological stress and inflammatory burden.

Several recent studies have investigated the prognostic utility of LAR in patients with MI, yet their findings remain inconsistent - particularly regarding its predictive accuracy across different MI subtypes (ST-elevation myocardial infarction (STEMI) vs. non-ST-elevation myocardial infarction (NSTEMI)), the presence of cardiogenic shock, and varying cut-off values [8-10]. Additionally, there is no standardized approach to the timing of LAR measurement, or uniform consensus on its interpretation, which presents a barrier to routine clinical implementation [11]. Variability in laboratory assays, differences in albumin/lactate kinetics, and lack of clear thresholds further complicate its clinical adoption.

Given these uncertainties, a systematic review and meta-analysis are both timely and necessary to synthesize current evidence, address heterogeneity in findings, and evaluate the robustness of LAR as a prognostic tool in MI. This review also aims to explore subgroup differences - such as between STEMI and NSTEMI - and discuss the clinical feasibility of integrating LAR into risk assessment protocols, considering real-world constraints like assay availability, timing, and interpretability across diverse populations. This systematic review and meta-analysis aim to evaluate the prognostic value of LAR at admission for predicting all-cause mortality in patients with MI. By consolidating existing data, we aim to clarify its potential role in clinical risk stratification and highlight practical considerations for its use in contemporary cardiovascular care.

## Review

Methodology 

Literature Search 

A comprehensive literature search was conducted to identify studies evaluating the prognostic value of the LAR at admission for predicting mortality in patients with MI. The search was performed across three major electronic databases: PubMed, Embase, and Web of Science, along with Google Scholar. The search period was limited to studies published from inception to March 10, 2025. To ensure completeness, the reference lists of included studies and relevant reviews were also manually screened for additional eligible articles. The search was performed independently by two authors. Any disagreement that arose during this process was resolved through discussion. 

*Search Strategy* 

The search strategy combined Medical Subject Headings (MeSH) and free-text keywords related to MI, lactate, albumin, and mortality or prognosis. The following MeSH terms were used: "Myocardial Infarction" (MeSH), "Lactates" (MeSH), "Albumins" (MeSH), "Prognosis" (MeSH), and "Mortality" (MeSH). Free-text keywords included variations such as "myocardial infarction," "heart attack," "acute myocardial infarction," "AMI," "lactate-to-albumin ratio," "lactate/albumin ratio," "LAR," "serum lactate," "serum albumin," "mortality," "death," "survival," "prognosis," "predictive value," and "risk stratification." Boolean operators (AND, OR) were used to combine terms appropriately. Filters were applied to restrict results to human studies published in the English language. The full search strategy was adapted for each database to ensure sensitivity and comprehensiveness in capturing all relevant studies.

Eligibility Criteria and Study Selection 

Studies were included if they met the following criteria: (1) observational cohort or case-control design; (2) adult patients (≥18 years) with a confirmed diagnosis of MI; (3) LAR measured at admission; and (4) mortality (in-hospital or long-term) reported as an outcome. Studies were excluded if they: (1) involved pediatric patients; (2) did not report LAR values or mortality outcomes; (3) were case reports, editorials, reviews, or conference abstracts; or (4) were duplicate publications. 

Two independent reviewers screened the titles and abstracts of all retrieved records. Full texts of potentially relevant studies were reviewed to assess eligibility based on the predefined criteria. Disagreements between the reviewers were resolved through consensus or by consulting a third reviewer. Study selection was recorded using a PRISMA flow diagram.

Data Extraction 

Data extraction was performed independently by two reviewers using a standardized data collection form. Extracted data included study characteristics (author, year, country, study design, and sample size), details of LAR measurement (whether estimates were available as continuous or categorical, along with cut-off values). Adjusted and unadjusted risk estimates (e.g., hazard ratios (HRs) and odds ratios (ORs)) with 95% confidence intervals (CIs) were also extracted. If data were missing or incomplete, the corresponding authors were contacted for clarification. The primary outcome of interest was all-cause mortality, which included both short-term (in-hospital or 30-day) and long-term mortality (beyond 30 days) following MI. Studies reporting mortality outcomes were pooled for meta-analysis using appropriate statistical methods. The prognostic value of LAR was assessed using pooled effect estimates. 

Data Analysis 

Data analysis was conducted using RevMan version 5.4.1 (Cochrane, Oxford, United Kingdom). To assess the effect of high LAR on all-cause mortality, we calculated the HR with a 95% CI. A p-value of less than 0.05 was considered statistically significant. A random-effects model was used to calculate pooled estimates, to account for variations among the studies. Heterogeneity was assessed using the I² statistic, with an I² value of 50% or higher indicating significant heterogeneity. Sensitivity analysis was performed by removing one study at a time, and the findings were presented in a table.

Results 

**Figure 1:**
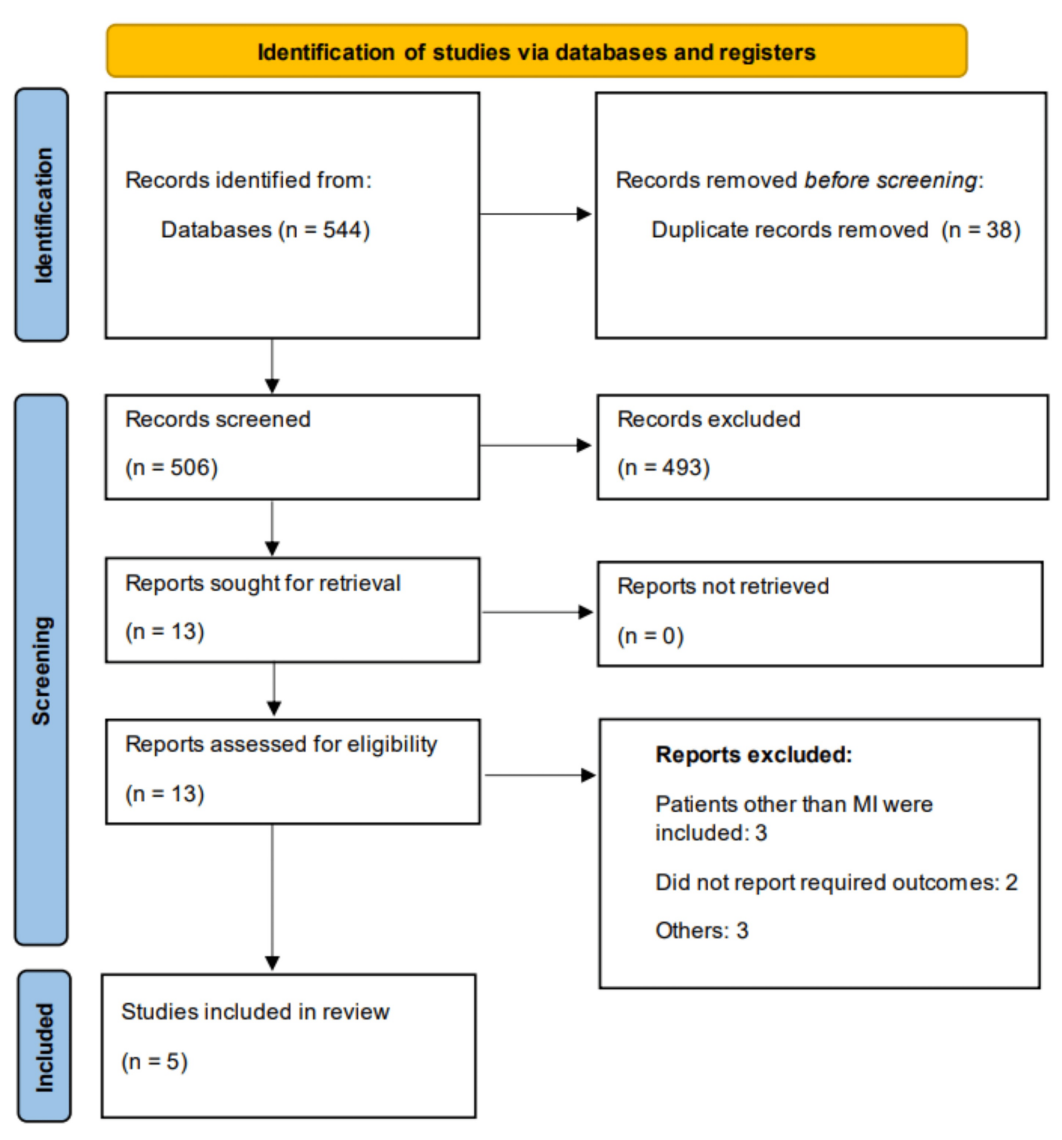
PRISMA flowchart showing study selection process

Figure 1 shows the PRISMA flowchart of the study selection process. Through online database searching, we found 544 studies. Duplicates were removed, followed by initial screening of eligible studies. Thirteen studies were chosen for full-text eligibility. Finally, five studies were included in this meta-analysis, enrolling 8,408 subjects with MI. Table 1 presents the characteristics of the included studies. All included studies were conducted in China and published between 2022 and 2024. Table 2 presents the quality assessment of the included studies.

**Table 1:** Study characteristics (n = 5)

Author	Year	Region	Follow-Up	Sample Size	Number of Deaths (n)	Males (n)
Chen et al. [12]	2023	China	30 days	2,626	305	1,734
Chen et al. [13]	2024	China	In hospital	2,816	204	1,765
Jin et al. [11]	2024	China	28 days	989	171	436
Wang et al [14]	2023	China	28 days	1,134	174	770
Zhu et al. [15]	2022	China	30 days	843	205	548

**Table 2:** Quality assessment of included studies using New-Castle Ottawa Scale (NOS)

Author	Selection (Total: 4)	Comparison (Total: 2)	Exposure/Outcome Assessment (Total: 3)	Overall Grade
Chen et al. [12]	3	2	3	Good
Chen et al. [13]	4	2	2	Good
Jin et al. [11]	3	1	3	Good
Wang et al. [14]	4	2	3	Good
Zhu et al. [15]	4	2	3	Good

Effect of High LAR on All-Cause Mortality

We included five studies assessing the effect of high LAR on all-cause mortality. Two studies assessed mortality rates up to 28 days, two studies followed patients for 30 days, while one study assessed in-hospital mortality rates. 

**Figure 2:**
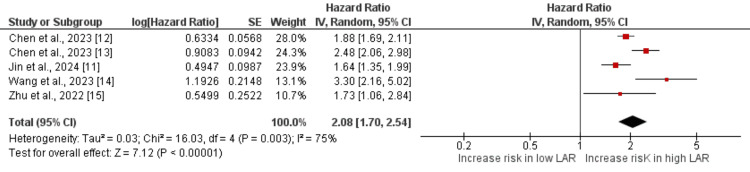
Association of LAR with mortality References [11-15] LAR: Lactate-to-albumin ratio

A pooled analysis of five studies showed that the risk of all-cause mortality was two times higher in patients with high-level LAR compared to their counterparts (HR: 2.08, 95% CI: 1.70 to 2.54), and the difference was statistically significant (p < 0.001), as shown in Figure 2. High heterogeneity was reported among the study results (I² = 75%).

We performed a sensitivity analysis by removing one study at a time, and the results are presented in Table 3. The pooled HRs remained consistent, indicating the robustness of the overall findings. The HRs ranged from 1.94 (95% CI: 1.62 to 2.33) to 2.24 (95% CI: 1.76 to 2.85) across the sensitivity analysis. The I² values showed moderate to substantial heterogeneity, ranging from 66% to 81%, suggesting that the between-study variability was not entirely explained by the removal of individual studies. This indicates that no single study had a disproportionate influence on the overall effect estimate, confirming the stability of the meta-analysis results.

**Table 3:** Results of sensitivity analysis HR: Hazard ratio; CI: Confidence interval

Author	HR	95% CI	I^2^
Chen et al. [12]	2.18	1.60 to 2.96	79%
Chen et al. [13]	1.95	1.58 to 2.42	66%
Jin et al. [11]	2.24	1.76 to 2.85	74%
Wang et al [14]	1.94	1.62 to 2.33	70%
Zhu et al. [15]	2.13	1.70 to 2.66	81%

Discussion 

This meta-analysis was conducted to determine the role of LAR in predicting the mortality of patients with MI. A total of five studies were included in this meta-analysis. A pooled analysis of these studies showed that LAR can serve as an independent predictor of all-cause mortality in MI patients, as risk was significantly higher in patients with high LAR. All five included studies reported an increased risk of all-cause mortality in the high LAR group, showing consistency in the effect of high LAR on prognosis in MI patients [11-15]. 

The association between LAR and poor outcomes in acute myocardial infarction (AMI) patients can be attributed to several interconnected mechanisms. Firstly, increased lactate levels indicate a shift to anaerobic metabolism, resulting from inadequate oxygen delivery to tissues - a hallmark feature of MI. Ventricular dysfunction, myocardial damage, and eventually poor clinical outcomes can result from tissue hypoperfusion and hypoxia [16]. Low albumin levels, which contribute to an elevated LAR, serve as an indicator of systemic inflammation. Pro-inflammatory cytokines can disrupt endothelial function, increase the risk of thrombosis, and worsen myocardial injury in patients with MI [17]. The coexistence of elevated lactate and reduced albumin reflects metabolic acidosis and impaired homeostasis. These metabolic imbalances can further deteriorate cardiac function and negatively impact the prognosis of MI [18].

LAR integrates nutritional and inflammatory factors, suggesting its importance in the relationship with outcomes in patients. LAR has been recognized as an important predictor of death, highlighting its function as a reliable prognostic indicator for patients who were admitted to an intensive care unit in hospitals in Germany due to sepsis from 2004 to 2009 [10]. According to a single-center retrospective cohort study, LAR is a more effective prognostic indicator for predicting in-hospital mortality among adult patients with sepsis than baseline serum lactate [19]. Previous research also reported that LAR was positively associated with all-cause mortality in patients with heart failure [20]. Ren et al. [21] reported that LAR showed a nonlinear relationship with in-hospital death among acute respiratory failure patients. 

Less data are available regarding the relationship between LAR and mortality in patients with AMI. This study offers evidence that LAR can be a robust predictor of death in MI patients. These results are especially important for MI, a disease that affects a large portion of the global population, because they allow for the early detection of individuals who are at high risk of passing away. This early detection is essential for maximizing therapeutic care and lowering the risk of further cardiac events.

In practical practice, patients with high LAR may require a different approach to treatment. First, customized therapeutic interventions can be used to meet particular clinical demands for critically ill MI patients. The prompt initiation of appropriate treatments, such as intra-aortic balloon pump (IABP) therapy for STEMI patients or optimal medical management (early or delayed percutaneous coronary intervention (PCI)) for NSTEMI patients, is made possible by the timely identification of high-risk patients. Second, heart failure, arrhythmias, and cardiogenic shock are among the sequelae that high-risk MI patients are susceptible to. By identifying this subgroup, targeted monitoring and proactive management of possible issues are made possible, which could have a major influence on the overall outcomes of patient care. For example, it is advised to increase the use of diuretics to reduce volume overload.

This variability in cut-off values may partially explain the high heterogeneity observed in our pooled analysis (I² = 75%). The differences likely reflect variations in patient populations, laboratory methodologies, timing of measurements, and institutional practices across studies. It is worth noting that all included studies determined their cut-off values using data-driven approaches (primarily receiver operating characteristic (ROC) curve analysis) rather than pre-established thresholds, which further contributes to this variability. Future prospective studies should focus on establishing standardized LAR measurement protocols and validating optimal cut-off values across diverse patient populations. Additionally, the integration of LAR into existing risk prediction models, such as the GRACE (Global Registry of Acute Coronary Events) or TIMI (Thrombolysis in Myocardial Infarction) scores, may enhance its clinical utility by providing incremental prognostic information beyond traditional risk factors. 

Study limitations

This meta-analysis has several limitations that should be acknowledged. Firstly, only five studies were included, and all were retrospective in design, which introduces a risk of selection and confounding bias. To minimize the impact of confounding bias, we used adjusted HRs from four of the five studies, all of which demonstrated a significant association between high LAR and increased mortality. Secondly, we were unable to conduct subgroup analyses based on key variables that could potentially influence outcomes, such as age, type of procedure, and comorbidities, due to limited data availability. Thirdly, long-term mortality could not be assessed because of the absence of sufficient long-term follow-up data across the included studies. Additionally, all included studies were conducted in China, which limits the generalizability of the findings to more diverse populations. Moreover, we were not able to perform a publication bias analysis because the included studies were less than 10.

Despite these limitations, the findings of this meta-analysis provide important insights into the prognostic value of LAR in patients with MI. The consistent association between high LAR and increased mortality highlights the potential utility of LAR as a simple and accessible biomarker for early risk stratification in clinical practice. Future research should focus on conducting large-scale, prospective studies to validate these findings and explore the underlying mechanisms linking LAR to adverse outcomes in MI. 

## Conclusions

This meta-analysis suggests that the LAR may serve as a promising prognostic biomarker in patients with MI. By pooling data from five retrospective studies involving 8,408 patients, we observed a consistent association between elevated LAR at admission and increased risk of all-cause mortality. Although most studies reported adjusted estimates, the methodological heterogeneity - including variability in adjustment models - warrants cautious interpretation. These findings highlight the potential of LAR as a cost-effective and easily accessible indicator that captures both metabolic and inflammatory stress. However, due to the retrospective nature of the included studies and their geographic limitation to China, further high-quality prospective research is necessary to validate these findings in more diverse populations. LAR should not yet be considered a standalone tool for clinical decision-making, but rather a potential adjunct to established risk scores such as GRACE or TIMI, especially in subgroups with high inflammatory burden or metabolic derangements. Ultimately, the integration of LAR into personalized risk assessment frameworks could enhance early prognostication and guide targeted management in MI - pending external validation and standardization of measurement protocols.
